# Low mother-to-child HIV transmission rate but high loss-to-follow-up among mothers and babies in Mandalay, Myanmar; a cohort study

**DOI:** 10.1371/journal.pone.0184426

**Published:** 2017-09-08

**Authors:** Khine Wut Yee Kyaw, Myo Minn Oo, Nang Thu Thu Kyaw, Khaing Hnin Phyo, Thet Ko Aung, Theingi Mya, Nilar Aung, Htun Nyunt Oo, Petros Isaakidis

**Affiliations:** 1 Department of Operational Research, International Union Against Tuberculosis and Lung Disease (The Union), Mandalay, Myanmar; 2 HIV unit, International Union Against Tuberculosis and Lung Disease (The Union), Mandalay, Myanmar; 3 Department of Obstetrics and Gynecology, Central Women Hospital, Mandalay, Myanmar; 4 Neonatology unit, Central Women Hospital, Mandalay, Myanmar; 5 National AIDS Program, Department of Public Health, Ministry of Health and Sports, Nay Pyi Taw, Myanmar; 6 Department of Operational Research, Médecins Sans Frontières (MSF) / Doctors Without Borders, Mumbai, India; Azienda Ospedaliera Universitaria di Perugia, ITALY

## Abstract

**Introduction:**

Loss-to-follow-up (LTFU) throughout the Prevention of Mother-To-Child Transmission (PMTCT) cascade remains one of the major threats to the success of PMTCT programs. In this study, we aimed to determine the mother-to-child transmission rate in a programmatic setting and to determine factors associated with LTFU among enrolled mothers and unfavorable outcomes among HIV-exposed babies which includes being HIV positive, death and LTFU.

**Methods:**

A retrospective cohort study reviewing routinely collected data in an Integrated HIV care program, Mandalay, Myanmar in June 2016.LTFU means mother/infant missing appointed visit for more than three months.

**Results:**

Of 678 pregnant women enrolled in PMTCT program between March 2011 and June 2014, one stillbirth and 607 live births were recorded in this cohort. Of 457 HIV-exposed babies with HIV-test recorded at the end of the intervention, nine (2%) were HIV-positive. Pregnant women’s and exposed-babies’ LTFU rate was 7 per 1000 person-years, and 10 per 1000 person-years respectively. PMTCT option B protocol was found to be significantly associate with maternal LTFU [adjusted Hazard Ratio (aHR) 95% CI: 3.52 (1.38–8.96)] when compare to mothers receiving option B+/lifelong antiretroviral therapy (ART). Weight <2.5 Kg at enrolment, receiving mixed-feeding, vaginal delivery and option B PMTCT protocol were significantly associated with unfavorable outcomes among exposed babies [aHR(95% CI): 5.40 (1.66–17.53), 5.91(1.68–20.84), 2.27 (1.22–4.22) and 2.33 (1.16–4.69) respectively].

**Conclusion:**

Mother-to-child HIV transmission rate in this public hospital-based program was lower than the 5% national target, which indicates a successful PMTCT intervention. However, a high proportion of HIV-infected mothers and exposed babies LTFU was recorded. Lifelong ART provision to HIV-positive pregnant women was shown to reduce exposed babies’ LTFU, death and transmission rate (unfavorable outcomes) in this setting. Lessons learned from this program could be used to inform policy and practice in the country, while the programmatic challenge of LTFU should be urgently addressed.

## Introduction

Mother-to-child transmission (MTCT) of human immunodeficiency virus (HIV) remains a major health problem worldwide even though the rate of transmission globally fell from 26% in 2009 to 16% in 2013 and 10% in 2015[1]. The transmission can happen during pregnancy, delivery and breast feeding. It depends upon different factors including acute HIV infection in mothers, high maternal viral load, low CD4-cell counts, vaginal delivery, prolonged rupture of fetal membranes, breastfeeding, no antiretroviral therapy (ART) or short duration of ART before delivery and poor adherence to treatment. With a comprehensive interventions the risk of MTCT can be reduced to less than 5% (or even lower) in breastfeeding populations from a background risk of 35%, and to less than 2% in non-breastfeeding populations from a background risk of 25%[2–9]. The annual number of new infection among children was reduced globally by 56% since 2010 and 70% since 2000 by improvement of PMTCT intervention.[1]

In 2013, 54% of pregnant women in low and middle-income countries did not receive routine HIV testing during antenatal care (ANC), which is a key step to accessing HIV prevention, treatment and care[10]. In 2015, 300,000 women did not receive ART or antiretroviral (ARV) prophylaxis to prevent MTCT. There is also high rate of treatment drop out in women who are pregnant and breastfeeding[1]. Globally, WHO and UNICEF projected that 1.9 million of children will acquire HIV infection in 2020[11].

In Myanmar, HIV prevalence in the adult population aged 15 years and older was estimated at 0.54% in 2014; it has considerably declined from 0.94% in 2000[12,13]. The HIV prevalence among pregnant women attending ANC was stable at less than 1% from 2011 to 2013[13]. At the end of 2013, about 3,000 women received PMTCT services, and nearly 90% of them were put on ART or ARV prophylaxis[14]. Myanmar has set the target in the National Strategic Plan to reduce the transmission rate to <5% by 2020 and to eliminate mother to child transmission of HIV by 2025.[15]

The PMTCT program in Myanmar is routinely monitored and evaluated however there has been no in-depth analysis of outcomes and factors associated with successful prevention, neither has been documented on lessons learned that could inform policy and practice.

The aim of this study was to determine the mother-to-child transmission rate among HIV-infected mothers and their exposed babies enrolled in the Mandalay Central Women Hospital PMTCT program between March 2011 and June 2014 and to investigate the factors associated with LTFU among enrolled mothers as well as factors associated with unfavorable outcomes among HIV-exposed babies.

## Methods

### Ethics approval

Permission for the study was obtained from National AIDS Program, Myanmar. Ethics approval was obtained from the Ethics Review Committee at Department of Medical Research, Yangon, Myanmar and the International Union Against Tuberculosis and Lung Disease (The Union) Ethics Advisory Group, Paris, France.

### Study design

This was a retrospective cohort study using routinely collected data on PMTCT of an Integrated HIV Care (IHC) Program in Myanmar.

### Setting

Myanmar is Southeast Asian country bordering India, Bangladesh, China, Laos and Thailand. The population of Myanmar was 53,259,000 in 2013 with 33% of the population living in urban areas.[16] About 6 million people live in Mandalay Region which is located in the center of the country. The Central Women Hospital in Mandalay is the largest facility offering comprehensive PMTCT services in the region. Minority of HIV-infected mothers from outside Mandalay Region were also enrolled in this PMTCT program.

#### PMTCT program description

Since 2011, The Union has been implementing PMTCT services in Central Women Hospital in Mandalay as a PMTCT clinic under IHC program, in collaboration with the National AIDS Program (NAP) and public hospitals and clinics under the Ministry of Health and Sports (MoHS). The PMTCT clinic enrolled HIV-infected pregnant women as well as post-partum women along with their exposed babies mainly delivered at CWH also other public hospitals and private clinic, and provided comprehensive PMTCT services. This integrated PMTCT care is delivered by obstetricians and pediatricians from CWH and physicians from Mandalay General Hospital as well as medical officers employed by the Union. Three counselling sessions on adherence to ART and regular follow up are offered to PMTCT women (1^st^ session- at enrolment visit, 2^nd^ during the blood collection day, and 3^rd^ at a clinic appointed day) by peer counsellors and medical social workers from the hospital. The program collaborates with an extensive PLHIV network which provides comprehensive counselling services and LTFU tracing. The Union supports formula feeding to exposed babies until the 9^th^ month of age and provides counselling and demonstration on formula feeding preparation in collaboration with the medical saffs from the hospital. Last, all enrolled mothers and babies are followed at the CWH PMTCT clinic until 18^th^ month post-partum or until the child HIV status have been confirmed. Early infant diagnosis (EID) is also provided to HIV exposed infants.

HIV-infected pregnant women who were eligible for treatment were offered antiretroviral therapy (ART) for life if their CD4-counts is lower than cutoff point (CD4-count cutoff was 350 cells/mm^3^ before 2015 and 500 cells/mm^3^ after 2015)[3].Women with CD4-count higher than above cutoff point who did not need treatment for their own health were given antiretroviral (ARV) prophylaxis as per the following PMTCT protocols;

1. PMTCT option A (prior to 2013): women received AZT only
2. PMTCT option B (from 2011 to 2014): women received triple ART, during pregnancy, delivery and discontinued one week after breast feeding was stopped
3. PMTCT option B+ (2014 to date): women received triple ART during pregnancy, delivery and continued for life[2,17].

At enrollment, every mother and child are given a unique registration code. Baseline demographic characteristics along with history of pregnancy and delivery are collected and CD4-count for the mother is tested. After delivery, mother and infant are followed-up until 18 months post-partum. The follow-up visit frequency varied depending upon the attending physician, feeding practices, the health status of the mother and the infant and distance of patients’ resident. In every follow-up visit, mothers are asked to bring along their infants. Infants receive HIV virological testing (DNA/RNA PCR essay) at the age of 4–6 weeks and 9 months, and are tested for HIV serology by using rapid tests (Determine HIV-1/2) at the age of 9 and 18 months. The indeterminate results at 9 months are confirmed with DNA/RNA assay between 9 and 18 months of age. HIV-positive infants were transferred out to Pediatric IHC clinic for ART initiation of ART and continuation of care. Table 1 shows the programmatic definitions used in this study.

**Table 1:** Programmatic definitions.

HIV-exposed infant	An infant who is born to an HIV-infected mother until HIVdiagnosis is made.
Mother to child transmission of HIV (HIV positive)	an event of an infant who tested positive by DNA/RNA PCR test at 6^th^ week of age or confirmed by DNA/RNA PCR test at 9^th^ month of age or HIV antibody test at 18^th^ month of age.
Regular follow up	mother/infant is alive and in care by the date of analysis
Lost-to-follow-up (LTFU)	mother/infant missing appointed visit for more than three months
Death	mother/infant death, regardless cause of death
Transfer out	mother/infant transfer out to other IHC clinic

### Patient population

HIV-infected mothers and their exposed infants enrolled in the PMTCT program and received PMTCT intervention in Central Women Hospital, Mandalay, Myanmar between March 2011 and June 2014.

### Sources of data, data variables

In every visit at the PMTCT clinic, medical doctors filled out the standardized PMTCT visit forms, and trained data staffs transcribed the paper-based data into the electronic database of IHC program after each clinic session.

Collected variables included: PMTCT protocol, CD4-count before delivery, baseline WHO staging, mode of delivery, place of delivery, pregnancy outcomes, employment status and mother literacy status, sex of exposed infants, infant weight at enrolment, and mode of feeding.

### Statistical analysis

Data were extracted from the electronic database of the IHC program and imported into STATA version 14.2 (College Station, TX). Data were anonymzed and de-identified prior to analysis.

The number and proportion of mother LTFU, HIV transmission and unfavorable outcome of exposed babies (defined as HIV-positive, death and loss to follow-up) were calculated. Rate of LTFU among mothers and of unfavorable outcomes among exposed babies were calculated with censoring on 30^th^ June 2016 for all exposed babies to have follow-up period up to 18 months of age. Cox proportional hazards models were used to determine the factors associated with mothers’ and exposed babies’ outcomes. P-value <0.05 was regarded as significant. A set of a priori variables based on knowledge from previous literatures such as PMTCT protocol, CD4 before delivery and mode of delivery were included as covariate in multivariable analysis to find the association with LTFU outcome of HIV-infected mothers. Weight at enrollment, mode of feeding, maternal PMTCT protocol, duration of ART and mode of delivery were used to find the association with unfavorable outcomes among exposed infants. [5,8,18–20] Transferred-out mothers and exposed infants were excluded from the models. Proportionality assumptions were tested using Schoenfeld residuals, log-log plots, and observed versus predicted survival plots. Statistical significance was assessed at 5% probability of type-1 error and 95% confidence intervals (95% CI) were estimated for attrition rates and hazard ratios.

## Results

**Fig 1:**
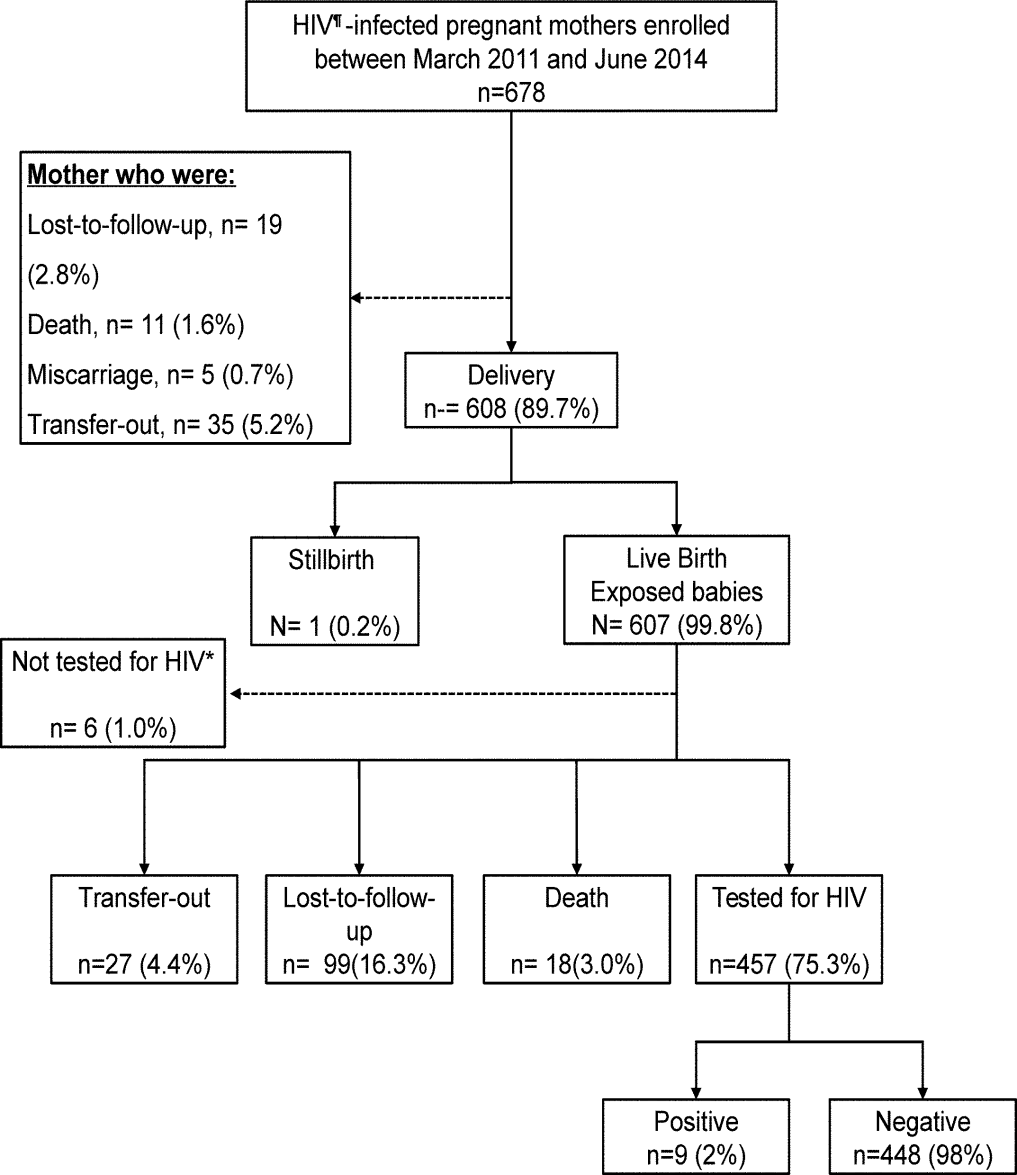
Cascade of PMTCT program in Central Women Hospital, Mandalay, Myanmar. HIV; ^**¶**^Human Immunodeficiency virus. *babies less than 9 months of age who were under regular follow-up and had not tested for HIV.

A total of 678 pregnant women were enrolled into the PMTCT program between March 2011 and June 2014. The median age (interquartile-range) at enrollment was 29 (26–33) years. Among these women, 19 (2.8%) were lost to follow-up before delivery, 1 (0.2%) had stillbirth and the remaining 607 (99.8%) live births. The LTFU rate among mother was 7 per 1000 person-years (PY) (95% CI 6–9). Among women who had delivery history (608), 12(2%) were death, 81 (13%) were LTFU, 497 (82%) were transferred out to other IHC sites after delivery and 18 (3%) were on regular follow-up by 30^th^ June 2016. Out of 607 HIV-exposed infants, 99 (16%) were lost to follow-up before the final diagnosis was made with LTFU rate of 10 per 1000 person-years (95% CI 8.52–12.9). Of 457 babies with HIV-test recorded 9 (2%) were tested HIV-positive, as shown in Fig 1.

Table 2 presents the socio-demographic and clinical characteristics of the enrolled mothers, rate of LTFU across different characteristics and their association with LTFU. Rate of LTF was highest in mothers who did not receive ART (44 LTFU in 1,000 PY follow-up) followed by option B and option A. The lowest LTFU rate was seen in mothers who received lifelong ART (4 LTFU in 1,000 PY follow-up). However, in adjusted analysis only maternal option B was found to be significantly associate with maternal LTFU (adjusted Hazard Ratio (aHR) 3.52, 95% CI 1.38–8.98).

**Table 2:** Characteristics and factors associated with loss to follow up among HIV-infected mothers enrolled in prevention of mother to child transmission program Mandalay, Myanmar, March 2011- June 2014.

Maternal characteristics	Total n	LTFU^¶^ n (%)	Rate in 1000 PY^#^ (95% CI)	Unadjusted HR^Ɣ^ (95% CI)	Adjusted HR^Ɣ^ (95% CI)
**Total**	678	101 (15)	7 (6–9)		
**Age group**					
15–24	123	20 (16)	8 (5–13)	1	
25–34	424	55 (13)	6 (5–8)	0.84 (0.47–1.48)	
≥ 35	131	26 (20)	9 (6–15)	1.17 (0.6–2.27)	
**PMTCT Protocol**					
No ART	60	28 (47)	44 (28–70)	9.81 (5.34–18.04)**	5.41 (0.65–45.23)
Option A	136	22 (16)	7 (4–11)	1.82 (0.98–3.39)	1.3 (0.46–3.74)
Option B	108	24 (22)	14 (9–22)	3.52 (1.99–6.23)**	3.52 (1.38–8.96)*
B+/Lifelong	374	27 (7)	4 (2–5)	1	1
**CD4 before delivery (cells/mm3)**					
<350	221	15 (7)	3 (2–5)	1	1
≥350	192	18 (9)	5 (3–7)	1.7 (0.82–3.53)	0.99(0.42–2.34)
Missing	265				
**WHO staging (Baseline)**					
I and II	550	89 (16)	7 (6–10)	2.42 (1.11–5.26)*	
III and IV	118	9 (8)	3 (1–6)	1	
Missing	10	3(0.3)			
**Pregnancy Outcomes**					
Alive	607	81 (13)	6 (5–8)	1	
Still Birth	1	0	-	-	
Miscarriage	5	1 (20)	23 (3–165)	4.25 (0.59–30.77)	
Missing	65				
**Mode of delivery**					
VD ^δ^	129	26 (20)	9 (6–14)	1.6 (0.94–2.72)	0.71 (0.25–2.05)
LSCS ^λ^	475	55 (43)	5 (4–7)	1	1
Missing	74	20 (27)			
**Place of delivery**					
Home	37	7 (19)	10 (5–23)	1.69 (0.73–3.9)	
Hospital	567	74 (13)	6 (5–8)	1	
Missing	74				
**Employment**					
No	361	46 (13)	6 (5–9)	1	
Yes	256	51 (20)	9 (7–13)	1.5 (0.97–2.33)	
Missing	61				
**Literate**					
No	38	8 (21)	11 (5–26)	1	
Yes	581	89 (15)	7 (6–9)	0.79 (0.32–1.95)	
Missing	59				

^¶^ LTFU; loss to follow up

* (p value < 0.05)

** (p value < 0.001)

^¥^PMTCT, prevention of mother to child transmission

^#^ PY, person-years

^Ɣ^HR, hazard ratio

^λ^ LSCS, Lower segment caesarean session

^δ^ VD, Normal vaginal delivery

Table 3 shows the infant and maternal socio-demographic and clinical characteristics, rate of unfavorable outcomes (HIV-positive diagnosis, LTFU and death) and their association with unfavorable outcomes of exposed babies. Rate of unfavorable outcomes was lowest in exposed infants whose mothers received longer duration of ART ≥24week before delivery (6 in 1000 PY follow up). In adjusted analysis, babies with weight <2.5 Kg at enrolment, receiving mixed-feeding, delivered by vaginal delivery and mother receiving option B PMTCT protocol were significantly associated with LTFU among exposed babies [aHR(95% CI): 5.40(1.66–17.53), 5.91(1.68–20.84), 2.27 (1.22–4.22) and 2.33 (1.16–4.69) respectively].

**Table 3:** Characteristics and factors associated with unfavorable outcomes (HIV+ diagnosis, death and loss to follow up) among HIV-exposed babies in prevention of mother to child transmission program, Mandalay, Myanmar, March 2011-June 2014.

Characteristics	Total n (%)	Unfavorable Outcome n (%)	Rate in 1000 PY^#^ (95% CI)	Unadjusted HR^Ɣ^ (95% CI)	Adjusted HR^Ɣ^ (95% CI)
**Total**	580	126 (22)	13 (11–16)		
**Sex**					
Male	304	53 (17)	11 (8–14)	0.83 (0.57–1.23)	
Female	257	55 (21)	13 (10–17)	1	
Missing	19	18 (95)			
**Weight at enrollment (kg)**					
≤2.5	11	7 (64)	54 (24–120)	5.03 (2.20–11.52)**	5.40 (1.66–17.53)*
>2.5	540	91 (17)	10 (9–13)	1	1
Missing	29	28 (97)			
**Feeding mode**					
Formula Feeding	489	83 (17)	10 (8–12)	1	1
Breast Feeding	71	32 (45)	34 (24–48)	3.45 (2.27–5.26)**	1.95 (0.94–4.10)
Mixed Feeding	10	3 (30)	24 (8–74)	2.46 (0.77–7.83)	5.91 (1.68–20.84)*
Missing	10	8 (80)			
**ARV prophylaxis**					
AZT^¥^	420	64 (15)	9 (7–12)	1	
NVP^€^	61	18 (30)	20 (13–32)	2.24 (1.32–3.8)*	
Missing	99	44 (44)			
**Maternal PMTCT Protocol**					
No ART	40	22 (55)	51 (32–82)	5.64 (3.21–9.91)**	
Option A	120	29 (24)	14 (9–20)	1.49 (0.93–2.42)	1 (0.48–2.1)
Option B	97	25 (26)	18 (12–27)	1.95 (1.20–3.18)*	2.33 (1.16–4.69)*
B+/Lifelong	323	50 (15)	9 (7–12)	1	1
**Maternal CD4 before delivery (Cells/mm3)**					
<350	213	37 (17)	9 (7–13)	1	
≥350	178	24 (13)	8 (5–12)	0.84 (0.49–1.44)	
Missing	189	65 (34)			
**Duration of ART before Delivery (week)**					
≤12	208	42 (20)	12 (9–16)	2.05 (1.07–3.93)*	1.68 (0.77–3.68)
12–24	112	17 (15)	8 (5–13)	1.39 (0.66–2.95)	1.43 (0.60–3.42)
>24	129	14 (11)	6 (3–10)	1	1
Missing	131	53 (40)			
**Maternal WHO staging (Baseline)**					
I and II	476	110 (23)	13 (11–17)	1.54 (0.88–2.70)	
III and IV	96	14 (15)	9 (5–15)	1	
Missing	8	2 (25)			
**Mode of delivery**					
VD ^δ^	124	44 (35)	25 (18–35)	2.49 (1.68–3.68)**	2.27 (1.22–4.22)*
LSCS ^λ^	452	80 (18)	10 (8–13)	1	1
Missing	4	2 (50)			
**Place of delivery**					
Home	36	15 (42)	31 (18–53)	2.54 (1.42–4.55)*	
Hospital	540	109 (20)	12 (10–15)	1	
Missing	4	2 (50)			
**Employment**					
No	314	67 (21)	13 (10–16)	1	
Yes	217	54 (25)	15 (12–20)	1.22 (0.84–1.78)	
Missing	49	5 (10)			
**Literate**					
No	31	8 (26)	19 (9–38)	1	
Yes	501	113 (23)	14 (11–16)	0.73 (0.35–1.49)	
Missing	48	5 (10)			

* (p value < 0.05)

** (p value < 0.001), HIV; Human Immunodeficiency Virus

^**#**^PY, person-years

^**Ɣ**^HR, hazard ratio

^¥^AZT, Zidovudine

^€^NVP, Neviriapine

^δ^ VD, Normal vaginal delivery

^λ^ LSCS, Lower segment caesarean session

## Discussion

This is the first study looking at the outcomes of HIV-infected mothers and their exposed infants enrolled in an integrated, hospital-based PMTCT program, in Myanmar. With a recorded mother-to-child HIV transmission risk of less than 2%, considerably lower than the 5% target set by the National AIDS Program, Myanmar. So this program could be considered successful at the national and regional level [15]. However, the high loss to follow-up observed among the pregnant women and their HIV-exposed infants highlights several programmatic challenges and requires urgent need to change in policy and practice.

The proportion of mothers who were LTFU after delivery was found to be five times higher than LTFU before delivery. There are many reasons of higher LTFU after delivery. The pregnant women have to attend ante-natal care clinics (ANC) regularly and therefore have less chance of dropping out from the PMTCT clinic during AN period which in this program is integrated into ANC and co-located in the same compound[21]. However, after delivery, mothers need to attend only one postal-natal clinic on 45^th^ days after delivery which may or may not be the same as PMTCT clinic appointment. Therefore, this is the possible reason that mother did not attend PMTCT clinic after delivery. In addition, most working women return to work soon after delivery, making regular PMTCT follow-up challenging and competing with other life priorities.

The LTFU rate in PMTCT IHC cohort was higher than adult IHC program cohort[22].This can lead to adverse health outcomes (higher morbidity and mortality) of mothers and their exposed babies. This is because the interruption of ART can lead to unsuccessful treatment outcomes in mother who required ART for their own health. In addition, MTCT risk can be increased if LTFU occurred before delivery as well as in breastfeeding mothers. Moreover, the exposed babies may miss the opportunity of timely diagnosis of HIV and early ART initiation if exposed babies were HIV positive. Disengagement from PMTCT program could be more likely to occur if mothers lived far from the hospital, if public transportation was not available, and if they were dealing with family and social problems: it is well documented that families affected by HIV/AIDS face several challenges from financial constrains to stigma and discrimination to loss of social resources and support[21,23,24].

In our study, pregnant women who were not on ART or were on option B PMTCT protocol were more likely to be LTFU compared to women on option B+ protocol (lifelong ART). Several different factors such as having later gestational age at ART initiation, type of PMTCT protocol (single does NVP), being newly diagnosed with HIV in the current pregnancy, stigma and discrimination, low knowledge of PMTCT program, low coverage of PMTCT programs, longer waiting time and provider clinical knowledge and attitude were associated with LTFU [9,21,24–26].

Exposed babies with body weigh less than 2.5 kg were more likely to experience unfavorable outcomes in this setting. This low weight at enrolment is associated with in utero transmission and pre-term delivery. In addition, vaginal delivery and mixed infant feeding were found significantly associated with unfavorable outcomes among exposed babies in this cohort; these factors were previously shown to be associated with such outcomes in other studies[8,18,27]. Exposed babies born to mothers who received no ART or received option A and B protocol were also more likely to have unfavorable outcomes; as ART was discontinued one week after breastfeeding cessation, we hypothesize that the mothers were less motivated to attend the clinic regularly and she also failed to come clinic for her exposed babies.

This study has several strengths. First, the routinely collected program data was used which reflect the real situation in the field; therefore the findings could give reasonable and programmatic feedback to the program. Secondly, the sample size in this study was relatively large as well as the completeness and robustness of the data compared to other PMTCT program descriptions in other countries[8,28–30]. Lastly, we strictly adhered to the Strengthening the Reporting of Observational Studies in Epidemiology (STROBE) guidelines in conducting and reporting the study[31].

The study did not come without certain limitations. Since this study used routinely collected data from an ongoing program, a certain amount of missing values were found and excluded from the analysis. Further strengthening of the monitoring and evaluation and reporting and recording systems is recommended. The reasons behind the LTFU of this programmatic cohort have not been explored in this study; further quantitative and qualitative studies were planned to conduct in order to address this most challenging programmatic outcome. Furthermore, the linkage between the antenatal HIV testing facility and PMTCT services were not looked at in this study where Lost to follow up was occurred up to 49% in low and middle income countries [26]. Lastly, some factors associated with LTFU of mothers and outcomes of exposed infants, variables found to be significant in unadjusted analysis did not hold when adjusted models were fit. The main reason for this could be the lack of statistical power.

## Conclusion

A low mother-to-child HIV transmission rate has been recorded in a public hospital-based program in Mandalay, Myanmar, which indicates a successful PMTCT intervention. There are several factors currently adopted in this setting such as integrated PMTCT care, counselling, LTFU tracing, provision of formula feeding and one stop services for both mothers and babies until confirmed HIV diagnosed of exposed babies, might have contributed to the overall success of this intervention, as evidenced by the low mother to child HIV transmission rate reported. According to the evidence produced by this study, we recommend that elements of the comprehensive package of this PMTCT program might be considered to be adopted by the National AIDS Program and expanded to other part of the country. However, high LTFU of HIV-infected mothers and exposed babies was also recorded. Lessons learned from this program could be used to inform policy and practice in the whole country, while the programmatic challenge of LTFU should be urgently addressed.

## Supporting information

S1 Table Characteristics and factors associated with three separate outcomes (HIV+ diagnosis, death and loss to follow up) among HIV-exposed babies in prevention of mother to child transmission program, Mandalay, Myanmar, March 2011-June 2014. * (p value < 0.05), HR, hazard ratio; ¥AZT, Zidovudine; €NVP, Neviriapine; δ VD, Normal vaginal delivery; λ LSCS, Lower segment caesarean session.
(DOCX)

S1 File Dataset for study on low mother-to-child HIV transmission rate but high loss-to-follow-up among mothers and babies in Mandalay, Myanmar; a cohort study. (DTA)
