# Supplementary material for: Low mother-to-child HIV transmission rate but high loss-to-follow-up among mothers and babies in Mandalay, Myanmar; a cohort study
Source: PLoS One. 2017 Sep 8;12(9):e0184426. doi: 10.1371/journal.pone.0184426 (PMC5590939; doi:10.1371/journal.pone.0184426)
Supplement: S1 Table — * (p value < 0.05), HR, hazard ratio; ¥AZT, Zidovudine; €NVP, Neviriapine; δ VD, Normal vaginal delivery; λ LSCS, Lower segment caesarean session. (DOCX) [file pone.0184426.s001.docx]

**S1 Table. Characteristics and factors associated with three separate outcomes (HIV+ diagnosis, death and Loss to follow up) among HIV-exposed babies in Prevention of mother to child transmission program, Mandalay, Myanmar, March 2011-June 2014.**

| **Characteristics** | Lost to follow up | | Dead | | | HIV positive | | |
| --- | --- | --- | --- | --- | --- | --- | --- | --- |
|  | HR (95% CI) | aHR (95% CI) | HR (95% CI) | aHR (95% CI) | HR (95% CI) | | aHR (95% CI) |  |
| **Total** |  |  |  |  |  | |  |  |
| **Sex** |  |  |  |  |  | |  |  |
| Male | **0.63 (0.41-0.95)** |  | 2.32 (0.74-7.29) |  | 1.64 (0.41-6.57) | |  |  |
| Female | 1 |  | 1 |  | 1 | |  |  |
| Missing |  |  |  |  |  | |  |  |
| **Weight at enrolment (kg)** |  |  |  |  |  | |  |  |
| ≤2.5 | **4.52 (1.83-11.16)** | 3.88 (0.93-16.17) | 6.47 (0.83-50.4) | **19.98 (1.99-200.4)*** |  | |  |  |
| >2.5 | 1 | 1 | 1 | 1 |  | |  |  |
| Missing |  |  |  |  |  | |  |  |
| **Feeding mode** |  |  |  |  |  | |  |  |
| Formula Feeding | 1 | 1 | 1 | 1 | 1 | | 1 |  |
| Breast Feeding | **2.86 (1.81-4.51)** | 1.75 (0.82-3.74) | **5.74 (1.82-18.08)** | 2.26 (0.34-15.3) | **10.47 (2.8-39.08)** | | 4.95 (0.11-25.47) |  |
| Mixed Feeding | 1.82 (0.45-7.45) | 4.19 (0.94-18.73) | 7.19 (0.88-58.73) | 7.6 (0.67-86.28) |  | |  |  |
| Missing |  |  |  |  |  | |  |  |
| **ARV prophylaxis** |  |  |  |  |  | |  |  |
| AZT¥ | 1 |  | 1 |  | 1 | |  |  |
| NVP^€^ | **1.98 (1.1-3.57)** |  | 2.09 (0.43-10.07) |  | **9.4 (2.1-42.16)** | |  |  |
| Missing |  |  |  |  |  | |  |  |
| **Maternal PMTCT Protocol** |  |  |  |  |  | |  |  |
| No ART | **5.98 (3.28-10.9)** |  | 4.16 (0.83-20.82) |  |  | |  |  |
| Option A | 1.3 (0.77-2.19) | 0.73 (0.33-1.62) | 2.66 (0.85-8.34) | 1.76 (0.26-11.8) | 0.38 (0.05-3.13) | |  |  |
| Option B | **2.15 (1.3-3.56)** | **2.1 (1.04-4.25)*** | 1.07(0.21-5.32) | 1.43 (0.12-16.68) | 0.72 (0.09-5.9) | |  |  |
| B+/Lifelong | 1 | 1 | 1 | 1 | 1 | | 1 |  |
| **Maternal CD4 before delivery (Cells/mm3)** |  |  |  |  |  | |  |  |
| <350 | 1 |  | 1 |  |  | |  |  |
| ≥350 | 0.77 (0.43-1.38) |  | 0.86 (0.23-3.25) |  |  | |  |  |
| Missing |  |  |  |  |  | |  |  |
| **Duration of ART before Delivery (week)** |  |  |  |  |  | |  |  |
| ≤12 | **2.37 (1.17-4.78)** | 2.09 (0.93-4.7) | 1.33 (0.34-5.21) | 1.19 (0.18-7.88) |  | |  |  |
| 12-24 | 1.4 (0.62-3.16) | 1.33 (0.54-3.32) | 0.38 (0.04-3.68) | 0.79 (0.06-10.13) | 0.83 (0-0) | | 0.84 (0.08-8.82) |  |
| >24 | 1 | 1 | 1 | 1 | 1 | | 1 |  |
| Missing |  |  |  |  |  | |  |  |
| **Maternal WHO staging (Baseline)** |  |  |  |  |  | |  |  |
| I & II | 1.82 (0.97-3.4) |  | 0.87 (0.25-3.09) |  | 0.87 (0.18-4.18) | |  |  |
| III & IV | 1 (0-0) |  | 1 |  | 1 | |  |  |
| Missing |  |  |  |  |  | |  |  |
| **Mode of delivery** |  |  |  |  |  | |  |  |
| VD ^δ^ | **2.3 (1.52-3.5)** | **1.97 (1.02-3.81)*** | **4.84 (1.62-14.4)** | **10.91 (1.84-64.59)*** | 2.99 (0.74-12.07 | | 3.42 (0.31-37.8) |  |
| LSCS ^λ^ | 1 | 1 | 1 | 1 | 1 | | 1 |  |
| Missing |  |  |  |  |  | |  |  |
| **Place of delivery** |  |  |  |  |  | |  |  |
| Home | 1.73 (0.84-3.57) |  | 14.64 (4.92-43.59) |  | 3.44 (0.42-28.12) | |  |  |
| Hospital | 1 |  | 1 |  | 1 | |  |  |
| Missing |  |  |  |  |  | |  |  |
| **Employment** |  |  |  |  |  | |  |  |
| No | 1 |  | 1 |  | 1 | |  |  |
| Yes | 1.06 (0.71-1.58) |  | **4.53 (1.46-14.06)** |  | 0.83 (0.2-3.48) | |  |  |
| Missing |  |  |  |  |  | |  |  |
| **Literate** |  |  |  |  |  | |  |  |
| No | 1 |  | 1 |  |  | |  |  |
| Yes | 0.86 (0.37-1.96) |  | 0.4 (0.09-1.77) |  |  | |  |  |
| Missing |  |  |  |  |  | |  |  |
